# Classical Sociology Through the Lens of Gendered Experiences

**DOI:** 10.3389/fsoc.2020.532792

**Published:** 2020-10-19

**Authors:** Anna Isaksson

**Affiliations:** School of Education, Humanities and Social Sciences, Halmstad University, Halmstad, Sweden

**Keywords:** classic sociology, sociology, educational content, gendered experiences, university education

## Abstract

There is a body of literature problematizing *the lack of* women's accounts in what is called classical sociology. However, limited efforts have been made to place female and male theorists' writings in juxtaposition with each other in order to demonstrate how their writings and theories differ. The aim of this article is to encourage discussion of how early female and male sociological theorists' descriptions and interpretations on the development of modern society were shaped by their own gendered experiences. Further, the aim is to shed light on the consequences this might provide for the teaching and learning of classical sociology. The article contributes a comparative analysis on how five authors, three female and two male, described and interpreted the transition from traditional to modern society through their gendered experiences. Their various interpretations illustrate how experiences are situated and that there is no complete and objective knowledge. As a consequence, universities should pay careful attention to gender distribution in their syllabi. Rather than achieve equal numbers of female and male authors, this will ensure that students are able to explore and understand classical sociology through the lens of different gendered experiences during their studies.

## Introduction and Background

In prologs and back-cover blurbs for classical literature in sociology, Karl Marx, Emilé Durkheim, and Max Weber are described as “pioneers,” “trailblazers,” and as the most significant social thinkers for understanding social life and societal development (e.g., Giddens, 1973; Morrison, 1995; Hughes et al., 2003; Calhoun et al., 2012). In several respects, these male classical scholars were pioneers in their fields. However, these men were not alone. Women were also major players in the development of sociological thinking and social theory. This was despite their work being largely invisible or “written out” of history as some authors suggest (Lengermann and Niebrugge-Brantley, 1998). Consequently, for several decades, it has been argued that higher education students are presented with sociology content based on only male voices. Approaching only a masculine view of classical sociological theories and concepts – that are presented as non-gender specific and universal – affects the conceptions of sociology as a science that students acquire (Stanley and Wise, 1993; Magdalenic, 2004, 2015). One way to address these issues is to incorporate the writings of female scholars into all syllabi in classical theory and thereby solving the “problem.” An equal representation of female and male authors could result in a more nuanced picture of the social world during the growth of modernity and issues such as politics, labor, and economics (c.f., Thomas and Kukulan, 2004). However, adjusting the syllabi to achieve gender balance, does not necessarily make students aware of how female and male scholars speak from their own situated experience and standpoint (c.f., Smith, 1987, 1999, 2005).

There is a body of literature problematizing *the lack of* women's accounts in classical sociology. Limited efforts have been made over time to place female and male theorists' writings in juxtaposition with each other to demonstrate how their writings and theories, due to their different experiences, differ from each other. However, some important contributions have been made in this field. Lengermann and Niebrugge-Brantley (1998) presented 15 women sociologists of the 19th and early 20th centuries. They described each woman's contribution to sociology and also some differences among these female sociologists and male sociologists concerning *their choice of aspects to be observed*. In their study, Grant et al. (2002) explored sociological writings by women and men between 1895 and 1940. They argued that women's work was different to that of men. More women than men wrote empirical, evidence-based articles. Further, women wrote about women, children, immigrants and the poor – subjects that tended to be rather absent in the men's work. Although contributions like these are valuable, this article argues that it is also important to provide university teachers and students with literature and articles that do not “only” describe differences in *what subjects* early female and male sociologists explored. More articles are needed that demonstrate how female as well as male sociological theorists interpreted the development of modern society *and the same subjects* very differently. Therefore, the aim of this article is to encourage discussion of how early female and male sociological theorists' interpretations of the development of modern society have been shaped by their own gendered experiences. Further, the aim is to shed light on what consequences this provides for teaching and learning classical sociology. Through a comparative analysis of five authors, three female and two male, this article documents how the authors describe and interpret some social processes and phenomena. Hence, the research questions that are explored are: How do these female and male scholars describe the transition from traditional to modern society through their lenses of gendered experiences? How did this societal development, according to these scholars, (re)shape gender and gender relationships?

The two questions reflect an interest in capturing how female and male theoreticians reflect on gender (or not) in their accounts of the development of modernity. Also, what happens to the teaching and educational content in classical sociology when both women and men “are permitted” to pursue it. As such, this article also engages in the wider debate of how to teach sociology and particularly *why* and *what* we should teach in sociology today (c.f., Harley and Natalier, 2013).

## The Selection of Themes and Authors

The writings of five authors (three women and two men) have been placed in juxtaposition with each other to demonstrate how early female and male sociological theorists' interpretations and further descriptions on the development of modern society are shaped by their own gendered experiences. Texts written by Charlotte Perkins Gilman, Jane Addams, and Marianne Weber are presented and discussed in relation to selected sections of Max Weber's and Emilé Durkheim's collective works. The selection of authors has been guided by the selection of themes. To capture different views of modernity's growth, a set of historical processes that brought the end of traditional society and replaced it with new forms of social orders were first identified. Societal changes in relation to religious beliefs, economic capitalism, urbanization, the division of labor, and new forms of social organizations are some processes that transformed the traditional order (Alexander et al., 2016). In this article, these processes were chosen and then highlighted through the writings of Charlotte Perkins Gilman, Jane Addams, Marianne Weber, Max Weber, and Emilé Durkheim. Several classical scholars, female as well as male, have written about these subjects and phenomena. However, these five theoreticians were chosen because they were somewhat contemporary with each other. Since their interpretations clearly differ, it can be argued that the juxtaposition can serve as a pedagogical tool to demonstrate how gendered experiences matter.

This article is organized as follows. In the first section, *The protestant ethic and the spirit of patriarchy*, the relationship between religious values and the rise of modern capitalism is explored from female and male scholars' perspectives. In the following section, *Men and society*, the female and male scholars' writings about social activities point to diverse understandings of the purposes and outcomes of social organization for women and men in modern society. The next section, *The elementary forms of the isolated life*, describes how female and male scholars interpreted the growth of modernity, and phenomena such as differentiation, specialization, and the division of labor, very differently. Finally, the last section concludes the article with some final remarks in relation to the aim and research questions.

## The Protestant Ethic and the Spirit of Patriarchy

*The relationship between religious values and the emergence of the spirit of modern capitalism is well-explored in the writings of Max Weber and Marianne Weber. In this section, it is demonstrated how they interpreted the consequences of this development in various ways. While Max Weber acknowledged religion as a catalyst for rationality and modern capitalism, Marianne Weber recognized how the protestant ethic and modern capitalism reinforced patriarchy*.

### Religion as a Catalyst for Rationality and Modern Capitalism

In his well-known study of religion, *The Protestant ethic and spirit of capitalism*, Max Weber demonstrates how the ethics of ascetic Protestantism played a crucial role in the development of modern society and capitalism. According to Max Weber, the Protestant ethic concerned the religious cornerstones that primarily influenced Calvin's profession of faith. The core of this profession of faith (the doctrine of predestination) was that God had decided that some people were predestined for life everlasting and others for everlasting death. For believers, this profession of faith caused constant worry. Who was chosen? In striving to ensure one's own salvation, believers tried to find signs from God. Hard work and self-discipline should contribute to a successful life, which in turn could be a sign from God that one was chosen (Weber, 1904-5/2007:233ff). The rational way of life on the basis of the idea of a calling was accordingly an important driver of capitalism. The spirit of modern capitalism should therefore, according to Weber, be understood as a part of the development of rationalism as a whole. However, Weber was very doubtful about the rationalization of the world. For him, cold calculation would push away value aspects and make the world boring. The only area, which to some extent was freed from the so-called iron cage of rationality, was love and sexual passions (Weber, 1949).

What distinguishes Weber's analysis of the Protestant ethic and spirit of capitalism, in relation to societal development and the growth of modernity, is a gender-neutral terminology. However, it could be argued that gender very much permeates Weber's thinking and observations. In *The Protestant Ethic and the Spirit of Capitalism*, Weber implicitly demonstrates that the masculinity that was favored in western capitalism was related to participation in the public sphere in collaboration with other men. This is a masculinity that builds on homosocial competition (c.f., Connell, 2005). Weber emphasizes how important it was for a *man* to belong to a church or association where ethics and morals were asserted as important aspects to succeed in one's career. For example, Weber presents the story of a businessman who wanted to open a bank. The first thing the businessman did was to seek admission to the Baptist movement. If the man was accepted into the congregation, it was seen as an absolute guarantee for a gentleman's ethical and moral qualities. The chances of succeeding as a businessman were markedly affected if one was recognized and legitimized by other men (Weber, 1904-5/2007).

Weber considers God's confirmation as hardly sufficient to improve the modern capitalist business ethos that was growing forth. Men's confirmation of each other, practiced through qualifying examinations in the voluntary societies/associations, was probably just as important as the idea of a calling in the establishment of the rational way of life. In other words, with the growing rationality and modernity, homosocial masculinity grows forth, something that Weber actually describes, but keeps from making into a question about gender relationships.

### Religion as a Catalyst for Women's Subordination

Max Weber provides an understanding of religion and implicitly homosocial masculinity as a catalyst for social action in the development of modern capitalism. Marianne Weber (1912/2003) describes religion as a catalyst for social action of a different nature. According to her, the Protestant ethic had a crucial influence over marriage as an institution. Religion sanctioned women's subordination, among other things, through reference to the Fall of Man:

New arguments in the Bible were sought for the subordination of the woman. Thus, Luther cites Eve's Fall from Grace very emphatically as a historical source: “If Eve hadn't sinned, she would have reigned together with Adam and ruled as his helper.” But now the Regime belongs to him alone, and she must bow before him as before her master (Weber, 1912/2003:88).

Women's subordination was also formulated as an expression for the wish of God. In addition, Marianne Weber demonstrates how Puritanism reinforced the idea of monogamy and encouraged men and women to strive for moral perfection. Marital sensuality was solely a means for reproduction in God's honor.

But, on the other hand, the spirit of Protestantism also contributed to the deepening of the marital ideal, and the shaping of everyday marital life. Namely, through those currents outside of the official churches of the Reformation that are classified as Puritan. Of course, Puritanism made a detour that is not easily recognizable. It, namely, carried into the world and into the institution of marriage with inexorable strictness the ascetic ideals of monasticism: rejection of all life pleasures and suppression of sensuality. Luther's God had still, just like the Catholic God, in magnanimous generosity turned a blind eye toward marital sensuality. The God of the Puritans allowed marital sensuality only for the purpose of the procreation of children for the greater glory of God (Weber, 1912/2003:88).

So, in parallel with the growth of industrialization, Marianne Weber (Weber, 1912/2003:96) argues that Puritanism transformed sexual activities to religiously meaningful and highly disciplined tasks. According to Marianne Weber, modernity began as a positive process. Before industrialization, women's relationships were limited to family and kinship relationships. Women's identity and awareness were especially oriented toward the best interests of the man and the children. With modernity, however, potential forces were born that could break women's limited interactions and isolation in the home. New institutions produced ideals that could contribute to intellectual emancipation among women and challenge the nature of marriage. However, according to Marianne Weber, when religion and capital joined forces in common maxims, these ideas were manipulated in the interest of the patriarchy. With the industrial format that grew forth, capital gained from women's subordination and continued (unpaid) work in the private sphere (Weber, 1912/2003:101).

In sum, Marianne Weber's social analysis illustrates that the Protestant ethic not only contributed to growing rationality in the sense of industrious and hard work in the spirit of capitalism. The marital ideal was also rationalized in the interest of capital. What Max Weber views as the rational iron cage's final bastion – love and marriage – is described by Marianne Weber as perhaps the most rational from a capitalist perspective. Marianne Weber introduces gender asymmetrical perspectives and makes women and the private sphere visible in a way that Max Weber does not. Even if women are not particularly present in Max Weber's ideal-typical discussion about the Protestant ethic and the spirit of capitalism, it could be argued that gender is implicitly present. Max Weber describes how masculine affirmation strategies and homosocial masculinity positions grow forth with modernity. However, the ideals of masculinity and its consequences from a gender perspective are rather absent in Max Weber's analysis.

## Men and Society

*With modernity, modern capitalism was born. Charlotte Perkins Gilman as well as Max Weber write about how important it was for men to become members in social clubs and to have social relationships with other men in order to succeed as businessmen. This section shows how they interpreted the consequences of men's different ways of organizing themselves very differently. It also sheds light on how Max Weber's analysis does not take women's organization into account and how men's way of organizing themselves had negative consequences for women*.

### Social Organization as a Key Factor for Male Power and Female Subordination

The previous section demonstrated how Max Weber emphasizes the importance for a *man* to belong to a church or association. If a man was recognized and legitimized by other men, the chances of succeeding as a businessman increased. However, in the first volume of *Economy and Society*, Weber argues it is not only religious actions and beliefs that matter. Membership in a friendship society, a society for veterans of war and even a bowling club can be of utmost importance. It gives the man relationships that are beneficial far beyond the purpose of the association. Thus, the connections between finances and group activity were accordingly very much a rational connection. As Weber expresses it, membership in a group gave social prestige and financial benefit even if the interests the organization safeguarded were insignificant to the individual member. Men's ways of socially organizing themselves had a profound and positive (and even necessary) impact on their financial activities (Weber, 2019, see also Weber, 1914/2007).

Charlotte Perkins Gilman makes a different description and interpretation of the development of social clubs. In her book, *The Man-Made World*, Gilman points out the negative consequences that the ideal of masculinity and the male hegemony have for women, as well as prosperity and humanity as a whole. Put simply, when theorizing on a number of social phenomena and institutions such as economics, industry, politics, crime, education, sports, religion, literature, art, family, and health, Gilman Perkins (1914/2001:201) states that masculinity distorts humanity.

According to Gilman, women's isolated positions constrained their possibilities to organize themselves. When they organized themselves, they had entirely different issues than men to address on their agenda. In the so-called “women's clubs,” which began to form in modern society, participation was often motivated by the idea that women would “improve their minds.” Gradually, the clubs developed, and women began writing extensive reports on social affairs. What often characterized the clubs was that they strived to improve something – such as access to libraries, legal rights, or disadvantaged areas of poverty. Men did not need to do this in their clubs; the club activities could primarily serve as entertainment and relaxation. In the formal social channels and the institutions, men could carry out and achieve what they wanted (Gilman Perkins, 1914/2001:202). The men's clubs could also contribute to strengthening a man's legitimacy and credibility, which in turn were important for also implementing the social changes or economic activities a man intended to conduct. Put differently, the activities that concerned male contentment were also a part of the development of the homosocial ties that made it possible for men to carry out the financial projects and social changes they wanted to make. Women had to provide input from the sidelines, and their possibilities of influencing economics and society were limited.

In *Women and Economics*, Gilman also describes financial relationships in society and men's different ways of organizing themselves and acting collectively. As bachelors, men build up relationships that are built on friendship and equality. These relationships indeed change when men get married – they become more dependent on securing their income to secure their family's finances and living standard. Even when married men tend to become rivals, financial interests “force” them to organize themselves and build up relationships based on reciprocity. However, women become rivals for each other more through their isolated and antagonistic financial interests. They must always ensure that they have a man who can support them to ensure their own security. This leads to women in their “competition” not having anything to earn from organizing themselves and building up relationships corresponding to the men's (Gilman Perkins, 1898/2006:54-55).

In summary, Charlotte Perkins Gilman and Max Weber describe how different kinds of social clubs and organizations had an important impact over men's financial activities and the development of modern capitalism. However, in contrast to Gilman, Max Weber does not consider the negative consequences of this development for women. With modernity, the connection between women and isolation in the private sphere became as strong as the connection between men and society. With modernity, capitalism was born. This was a type of capitalism that had a cornerstone in financial and (homo)social power principles. Even though women organized themselves, the purposes and outcomes were different. Women organized themselves in order to achieve equal human rights rather than to earn money (c.f., Gilman Perkins, 1898/2006:25).

## The Elementary Forms of the Isolated Life

*The transition from traditional society to modern society was characterized by the division of labor and differentiation. In this section, it is demonstrated how Charlotte Perkins Gilman and Jane Addams describe this historical process as something that shaped social spaces for men and social isolation for women. While Gilman and Addams identify dramatic consequences for individual women as well as for the entire development of society, Durkheim regards this process as natural. According to him, women by nature did not have especially strong social needs and could withstand isolation significantly better than men*.

### The Division of Labor – Social Spaces for Men and Social Isolation for Women

As mentioned in the section “The protestant ethic and the spirit of patriarchy,” Marianne Weber was interested in the consequences of modernity on marriage as an institution. In the article *How Home Conditions React Upon Family*, Charlotte Perkins Gilman also demonstrates how marriage developed into a financial relationship when the husband realized what value the woman's unpaid work in the home had (Gilman Perkins, 1909:593). In her text *The Yellow Wallpaper*, she describes in novel form how the home becomes a women's prison and how being closed in could drive under-stimulated women to illness. However, even if it was social isolation that caused illness, women were considered, by psychologists, as having weak nerves by nature and thereby more easily develop hysteria (Gilman Perkins, 1997/1892). Gilman discusses how women's isolation in the private sphere had consequences for the women themselves. What she describes as the arrested womanhood did not tend to develop the best individuals. The isolated conditions and the limitations for women did not promote social progress as well (Gilman Perkins, 1997/1892). The foremost task for sociology must therefore, according to Gilman, be to see which structural changes are required to change marriage as an institution. The social orders that make the woman a property and a servant of the man must be counteracted. Breaking women's isolation would lead to more intellectual stimulation for women, which in turn would provide benefits in both the public and private sphere (Gilman Perkins, 1909).

In accordance with Gilman, Jane Addams describes how the growth of modernity through differentiation, specialization, and the division of labor resulted in greater participation for men in the public sphere, and isolation for women. Addams refers to women being isolated in one's own home. Paid work for women largely comprised of services that related to household work. According to Addams, what characterized this industry was that the employed women also lived with their employers. Addams considers this to be extremely problematic and she asked the question of why women, who prepared food and cleaned in another household, should not live somewhere else? Why should these women, like factory workers, not be able to come to their work in the morning and leave it in the evening? (Addams, 1896:538). Naturally, it had to do with the often merciless working conditions, with long working days and heavy household work, that the women were forced to accept. As demonstrated in the quote below, it was also related to notions that this industry could “take care” of the girls/women who did not have the capacity to contribute to what was considered to be the truly progressive industries in modern society.

She is belated in a class composed of the unprogressive elements of the community, and which is recruited constantly from the victims of misfortune and incompetence, by girls who are learning the language, girls who are timid and slow, or girls who look at life solely from the savings bank point of view (Addams, 1896:540).

The employers in this industry treated their workforce as servants in contrast to the other industries of modernity, where employers and employees were at such different hierarchical positions, without the employee being reduced to a servant. The women who worked in the factories could indeed have tough conditions, but in contrast to the maid, this woman participated in social life. The maid therefore ended up in an especially vulnerable and socially isolated position (Addams, 1896:544). As Addams expresses it in *Democracy and Social Ethics*:

She is obliged to live constantly in the same house with her employee, and because of certain equalities in food and shelter she is brought more sharply face to face with the mental and social inequalities (Addams, 1902/1988:44).

Addams did not see any change in sight as long as the maid's employer did not become aware of her almost non-existent ethical and moral principles (Addams, 1902/1988:49). She demonstrates that it is not only the maid's conditions that constituted a serious threat to both the private individual and society in general. Addams describes modern society as a society full of corruption. Capitalism has conquered morality. Capital bribes politics and politics bribes capital and money becomes the only motivational force as general morality, which everyone can relate to, becomes increasingly impossible (Addams, 1902/1988:103ff).

It could be argued that Addams' thoughts on the development of modernity are permeated by descriptions of the growth of homosocial masculinity. Like Max Weber, although more explicitly, she highlights that the masculinity that was favored in western capitalism was based on participation in the public sphere in collaboration with other men. What the alderman in the city demanded was loyalty from his subordinates, men who were good to him, men who stood behind his decisions and actions. Political life consisted of men who wanted to know that they were especially chosen and who were part of the group entrusted with political “gossip.” They wanted to belong to those who understood the nature of things and the order of the world. In their reasoning around this homosocial development, Addams refers to Mill who also believed that the man had a need for social contexts and a desire to make common cause with his “peers.” Paradoxically, the collective corrupt masculinity accordingly became synonymous with good morals. A man of high moral standards would think of himself not as an isolated individual, but as a part of a social organism. For the politically elected representatives, it was only important to convince the voters that (the politicians' own) individual needs were synonymous with “the best interests of the public” (Addams, 1902/1988:105).

In summary, Charlotte Perkins Gilman and Jane Addams show how society's differentiation created social spaces for men and social isolation for women. According to them, the division of labor had negative consequences for marriage as an institution.

### The Division of Labor and Differentiation – Different but Natural Roles Between the Sexes

In contrast to Charlotte Perkins Gilman and Jane Addams, Emilé Durkheim believed that the differentiation had created a functional complementarity between the sexes and a mutual dependence that according to him was the foundation for the family as an institution (Durkheim, 1893/2007). For Durkheim, it was hardly likely that women would ever be able to perform the same functions as men. Women could play important, but entirely different roles than men in society (Durkheim, 1897/2002). Durkheim describes that women by nature did not have especially strong social needs. He argued that women could withstand isolation and enclosure significantly better than men. In his text *Suicide*, he discusses this in detail. According to Durkheim, pious devotions and some pets to take care of are enough to completely meet the older unmarried woman's needs. The man, however, has a need for other activities. Since his social ego is more complicated and developed, he can maintain mental balance only if he finds new points of attachment beyond himself (Durkheim, 1897/2002). Durkheim thereby solves the problem of women's absence from the public sphere by referring to biology.

The division of labor was, according to Durkheim, necessary for larger society to develop. However, the collective conscience that characterized primitive and traditional society tended to decrease in modern society. In modern society, the degree of the division of labor is high and the collective conscience does not grow forth naturally. In this society, the individual is increasingly left alone. The result is too much freedom and too little morality. In order to sustain solidarity and morality, Durkheim advocates *human brotherhood*. According to Durkheim, this ideal was a guarantor for moral individualism and a strong modern society (Durkheim, 1893/2007).

It is easy to assume that Durkheim associates human brotherhood, collective conscience, and moral individualism primarily with the male gender. Firstly, brotherhood on a lexical level is naturally closer to the notion of it pertaining to relationships between men. Secondly, for Durkheim, women do not have social needs to the same extent as men and as society was not necessary for them for several reasons, Durkheim would probably realize that changes in the collective conscience did not affect women as strongly as they were not subjected to the same social strains as the men. Thirdly, if working life is the sphere of life that became the base for cohesion at the same time that morality was considered to be the most important principle of solidarity, the idea of the collective conscience should have its greatest source of development in working life. Mainly men were found there. Durkheim's ideal of human brotherhood as a positive force for counteracting the destructive consequences of the division of labor could again be interpreted as an encouragement and recognition of the necessity of homosocial masculinity.

In summary, Jane Addams saw clear disadvantages with the development that morality took in modern society. Human brotherhood, which in the spirit of Durkheim becomes a positive social force, is described by Addams in an opposite way. She described an increasingly corrupt male morality that had considerable consequences for the idea of the ethical and democratic society.

Addams and Durkheim differ not only in terms of their views on morality and the possible ways for modern society to handle the division of labor. It is also obvious that Durkheim provides a different description than what Addams and the other female scholars express above. Durkheim believed that women were somewhat well-suited to handle the isolation that the modern project entailed for them, by referring to the objective circumstances, the needs of the organism and women's less developed social needs. Public life and what happened on the production side were not very relevant for women. The female scholars provide a different picture. Women's isolation in the private sphere not only had dramatic consequences for individual women, but for the entire development of society. Accordingly, we are provided with different interpretations and explanatory models for the same phenomena. On one hand, we have Durkheim's analysis, on the other Addams' and Gilman's that partially harmonize with each other. If we read both women's and men's accounts of the development of modernity, we will have diametrically opposed views of “good morality,” the reasons for why women are shut away and the consequences of the elementary forms of isolated life.

## Discussion

The aim of this article has been to encourage discussion of how some early female and male sociological theorists' interpretations and further descriptions on the development of modern society are shaped by their own gendered experiences. Further, the aim has been to shed light on the consequences this might provide for the teaching and learning of classical sociology. In the article, a comparative analysis of five authors, three female and two male, has been performed to document how they describe some social processes and phenomena through the lenses of their gendered experiences. The following questions have been explored in the article: How do these female and male scholars describe the transition from traditional society to modern society through their lenses of gendered experiences? How did this societal development, according to these scholars, (re)shape gender and gender relationships?

### Divergent Accounts of the Same Phenomena

In the article, it has been clearly demonstrated how the female and male sociological theorists' interpretations and descriptions differ from each other as a consequence of their different standpoints and experiences. For example, in their descriptions of the transition between traditional society to modern society, women were placed in the private sphere and men were placed in the public sphere. However, the female and male scholars provide different descriptions of why this transition occurred and, above all, the consequences of the transition for humans and society. Gender and gender relations are to a great extent present in the female scholars' texts while the male scholars' reflections on gender is quite absent. Further, when gender is present in the male theorists' writings, the female subordination and women's role in society are naturalized and made rather unproblematic with references to capitalism, biology, and/or religion.

It is also obvious that the female and male scholars often highlight different explanatory models, aspects, and understandings regarding the same phenomena based on the gendered lenses they wear. As demonstrated above, the female and male scholars tend to have divergent accounts of phenomena such as religion, rationality, morality, the division of labor, capitalism, social clubs, and the consequences of modernity for women and men. For example, Marianne Weber's and Max Weber's respective descriptions of the origin and effects of the Protestant ethic and the spirit of capitalism represents one example of how women and men provide different descriptions of the same phenomenon, even though they lived in the same environment.

Max Weber discusses how the Protestant ethic contributed to growing rationality in the sense of industrious and hard work in the spirit of capitalism. Even though Max Weber does not use the term homo-sociality, he describes its development as a necessary factor in order to be given admission to the societies that affirmed a man's religious faith and thereby also his creditworthiness. The Protestant Ethic, resulting in growing rationality and forms of homo-sociality, was necessary for the capitalist development. However, it had consequences for the female subordination. These consequences are not theorized by Max Weber but by Marianne Weber who describes how the marital ideal was rationalized in the interest of capital. She argues that the Protestant ethic had crucial influence over marriage as an institution and religion sanctioned women's subordination. Women were, through religious arguments, obliged to the private sphere which was rational from a capitalist perspective. How homo-sociality strengthened men's positions in society is further developed by Charlotte Perkins Gilman in her reflections on women's and men's clubs. Max Weber, who described social clubs from his male perspective did not see any gender conflicts in relation to men's clubs. Men's clubs were, according to him, a rational way of strengthening a man's legitimacy, credibility, and homosocial ties which made it possible for men to carry out financial activities. However, Charlotte Perkins Gilman argues that men's homosocial ways of organizing themselves made it possible for men to earn money but also to make the social changes they wanted to make. Due to women's isolated positions, they had few possibilities of influencing economics and society.

In accordance with Charlotte Perkins Gilman, Marianne Weber and Jane Addams also wrote about how society's differentiation created social spaces for men and social isolation for women. They all describe how the division of labor had negative consequences for women and for marriage as an institution. When Durkheim advocates for the human brotherhood and describes it as a positive force for the good morality and for counteracting the possible destructive consequences of the division of labor – Jane Addams discusses disadvantages within the growth of the modern morality. Rather, she describes an increasingly corrupt morality that had negative consequences for the development of an ethical and democratic society. In summary, it is apparent that the female and male scholars provide us with different perspectives on the same phenomena and that gender and gender relations are present in different ways in the female and male scholars' texts.

### The Importance of Visualizing How Gendered Experiences Matter

It could be well-argued that these different interpretations and descriptions of the same phenomena described above, reflect that no one can have complete and objective knowledge. As Smith (1987; 2005) emphasizes, what one knows is affected by one's experiences and subject position in society. The female and male theorists chosen in this article demonstrate their advancement of theory through their various reflections on modernity. The results of the comparative analysis suggest that their different gendered locations shaped their theories. In other words, their gendered experiences shaped their understandings and the (normative) ways they were present and active in the creation of social theory and sociology. As women and men, they were socially situated in different ways. This made it possible for them to be aware of different things, to look at the world in different ways and to ask different questions.

Designing the content and context of teaching classical sociology based on the perspectives of both female and male scholars and further, contrasting their different descriptions of the transition from traditional society to modern society, should provide students with a broader view of sociology as a science. In addition, such an approach to teaching sociology introduces the significance of gender mechanisms in sociological analysis already in the teaching of classical sociology. This could also be done through an intersectional perspective, i.e., demonstrating how ethnicity, age, (dis)ability, sexual orientation, and class also shape interpretations and writings. In this article only white female and male scholars have been taken into account, which clearly has its limitations. Even though it provides the reader with insights about how gender matters, it also excludes other experiences and standpoints based on other power structures.

The sociology of the classics is often the first sociology content that students meet when they begin their sociology studies. Thus, which experiences and accounts that are presented as important for sociological analysis are of great significance concerning how students will come to view sociology as a science. An educational content of sociology based on various understandings provides students with a significantly multifaceted and nuanced sociology than the classical sociology traditionally taught in university institutions. Most importantly, visualizing different interpretations and descriptions and further, demonstrating and exploring them as expressions and consequences of gendered experiences underlines that all experiences are situated and that there is no complete and objective knowledge. As a consequence, universities should pay careful attention to the gender distribution in their syllabi.

Rather than achieve equal numbers of female and male authors, this will ensure that students are able to explore and understand classical sociology through the lens of different gendered experiences during their studies.

## Author Contributions

The author confirms being the sole contributor of this work and has approved it for publication.

## Conflict of Interest

The author declares that the research was conducted in the absence of any commercial or financial relationships that could be construed as a potential conflict of interest.
